# A timely reminder of technical limitations

**DOI:** 10.1111/1751-7915.12376

**Published:** 2016-06-24

**Authors:** James I. Prosser

**Affiliations:** ^1^ Institute of Biological and Environmental SciencesUniversity of AberdeenCruickshank Building, St. Machar DriveAberdeenAB24 3UUUK

Techniques, and our opinions of their value, can become set in stone. Initial development of techniques usually involves significant attention to optimisation, assessment of bias, testing in different situations, etc. This was certainly true for the first molecular techniques for characterisation of microbial communities in natural environments. In part this was due to good practise but the remarkable results obtained necessitated rigorous and careful testing to give confidence in the findings and counter unavoidable and justifiable scepticism.

As techniques become accepted and enter general use, there is less demand for testing, understandably. For example, it is well known that different DNA extraction methods introduce different biases when characterising microbial communities. However, microbial community ecology would advance very slowly if each study was required to include assessment and comparison of all available DNA extraction methods. The same applies to cultivation‐based studies, where isolates will be determined by the growth medium and cultivation conditions, but the full range of growth media cannot be used. These well‐known biases of cultivation‐based methods are frequently cited as justification for molecular techniques, but the biases associated with molecular techniques may be as important, but receive less critical attention.

Quantitative PCR (qPCR) is now commonly used to quantify genes in natural environments and is frequently used to estimate population abundance. It aims to determine the absolute abundance of genes in a sample, but few critical users of qPCR would claim that it provides an accurate measure of abundance. Cell lysis and DNA extraction are never complete and vary between phylogenetic groups and environmental samples. qPCR also relies on primers for target groups and is therefore subject to additional inaccuracies associated with primer coverage, specificity and bias.

The article by Musovic *et al*. highlights the importance of the design and choice of PCR primers, the significant differences in absolute abundance between qPCR assays with different primers and the need for careful assessment of their use in particular environments. It therefore provides a timely reminder that we should always be aware of the limitations of techniques, even those that are in common use. No technique, no protocol and no primer are perfect. This need not prevent use of techniques but their limitations and imperfections must never be forgotten when interpreting data and, most importantly, when designing a study and assessing whether experimental design and the techniques employed can achieve the aims of the study.

